# Cross-sectional associations between multisensory impairment and brain volumes in older adults: Baltimore Longitudinal Study of Aging

**DOI:** 10.1038/s41598-024-59965-w

**Published:** 2024-04-23

**Authors:** Chenxin Tian, Jennifer A. Schrack, Yuri Agrawal, Yang An, Yurun Cai, Hang Wang, Alden L. Gross, Qu Tian, Eleanor M. Simonsick, Luigi Ferrucci, Susan M. Resnick, Amal A. Wanigatunga

**Affiliations:** 1 Department of Epidemiology, Johns Hopkins Bloomberg School of Public Health, Baltimore, MD USA; 2 Center on Aging and Health, Johns Hopkins University, 2024 E. Monument Street, Suite 2-700, Rm 2-726, Baltimore, MD 21205 USA; 3 Intramural Research Program, National Institute on Aging, Baltimore, MD USA; 4 Department of Health and Community Systems, University of Pittsburgh School of Nursing, Pittsburgh, PA USA; 5 Department of Otolaryngology, Johns Hopkins School of Medicine, Baltimore, MD USA

**Keywords:** Sensory, Neuroimaging, Brain aging, Olfactory impairment, Epidemiology, Neuroscience, Cognitive ageing, Diseases of the nervous system, Sensory processing

## Abstract

Sensory impairment and brain atrophy is common among older adults, increasing the risk of dementia. Yet, the degree to which multiple co-occurring sensory impairments (MSI across vision, proprioception, vestibular function, olfactory, and hearing) are associated with brain morphometry remain unexplored. Data were from 208 cognitively unimpaired participants (mean age 72 ± 10 years; 59% women) enrolled in the Baltimore Longitudinal Study of Aging. Multiple linear regression models were used to estimate cross-sectional associations between MSI and regional brain imaging volumes. For each additional sensory impairment, there were associated lower orbitofrontal gyrus and entorhinal cortex volumes but higher caudate and putamen volumes. Participants with MSI had lower mean volumes in the superior frontal gyrus, orbitofrontal gyrus, superior parietal lobe, and precuneus compared to participants with < 2 impairments. While MSI was largely associated with lower brain volumes, our results suggest the possibility that MSI was associated with higher basal ganglia volumes. Longitudinal analyses are needed to evaluate the temporality and directionality of these associations.

## Introduction

Sensory impairment in the form of hearing and/or vision loss, proprioceptive impairment, lower vestibular function, and impaired olfaction is prevalent among older adults^1,2^. Among older Americans, an estimated 33% have hearing impairment^3^, 24% have olfactory impairment^4^, and 18% have vision impairment^3,5^. Importantly, prior research suggests that sensory impairments might be an early sign of cognitive decline^6,7^ and dementia^8^.

Studies have linked multiple sensory impairments (multisensory impairment [MSI]) to cognitive and physical decline^9,10^ and increased risk of mortality among older adults^11^. Emerging evidence show an association between single sensory impairments and altered brain structure^12,13^. Yet, whether the co-occurrence of these sensory impairments leads to lower volumes in each brain region or additional surrounding brain regions remains unclear. With neuroimaging evidence linking brain atrophy and cognitive change^14^, identifying sensory impairments or combinations of MSI associated with brain structure could provide novel mechanistic insights and modifiable or treatable targets involved between multiple sensory loss and cognitive impairment and dementia risk.

This study examined whether single and multiple sensory impairments are associated with relative brain volumes across several regions among cognitively unimpaired older adults. Though this study is exploratory, we hypothesized that a greater number of sensory impairments would be associated with smaller brain volumes and as a corollary larger ventricular space.

## Methods

### Participants

Participants were enrolled in the Baltimore Longitudinal Study of Aging (BLSA), an ongoing longitudinal cohort study conducted by the National Institute on Aging Intramural Research Program^15^. The BLSA recruits participants aged 20 years and older with no major physical or cognitive impairments or chronic diseases, except controlled hypertension. At each visit, participants undergo comprehensive physical, cognitive, sensory assessments, along with neuroimaging exams. Visits are scheduled every 1–4 years, depending on age (participants under age 60 visited every 4 years, those aged 60–79 years old visited every 2 years, and those aged 80 and above visited annually).

Eligibility criteria for this cross-sectional study include: (1) aged 50 years or older and (2) free of mild cognitive impairment or dementia based on a two-step assessment of neurocognitive testing. First, neurocognitive data of participants were analyzed, and cognitive impairment was defined if their Clinical Dementia Rating score were ≥ 0.5^16^ or if they had > 3 errors on the Blessed Information-Memory-Concentration test^17^. Second, the diagnosis of dementia and Alzheimer’s Disease were based on the *Diagnostic and Statistical Manual of Mental Disorders, 3rd ed., Revised*^18^ and the National Institute of Neurological and Communication Disorder and Stroke-Alzheimer’s Disease and Related Disorders^19^. Mild cognitive impairment was defined according to the Petersen criteria^20^.

**Figure 1:**
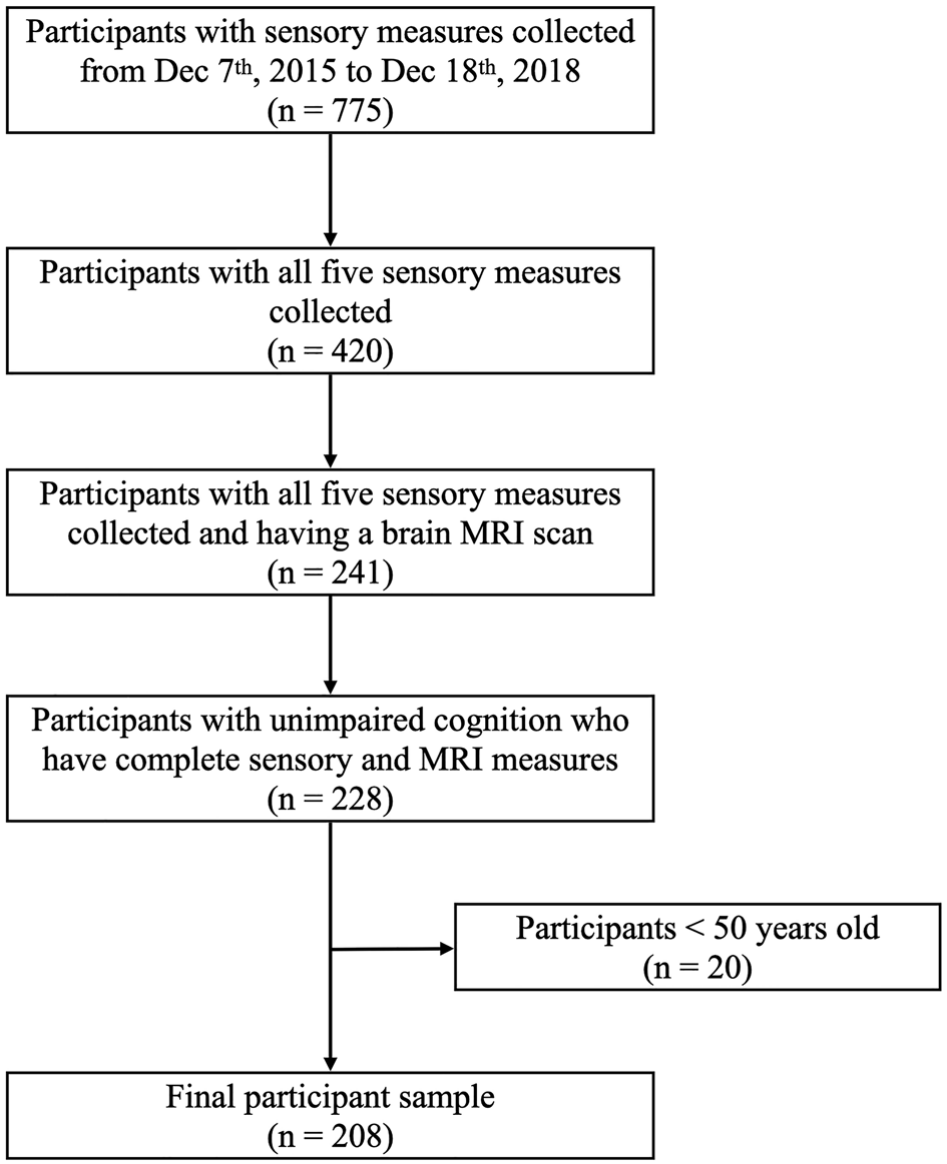
Baltimore longitudinal study of aging (BLSA) participant flowchart (n = 208).

A total of 775 participants had at least one of five sensory measures collected from December 2015 to December 2018 (Fig. 1). Approximately 54% had all five sensory measures collected at their most recent visit (n = 420). Along the 420 participants, 241 participants also had a brain MRI scan during the same visit. Thirteen participants were excluded due to cognitive impairments and twenty participants were excluded because they were younger than 50 years. The final analytic sample was 208 participants ≥ 50 years old who were cognitively unimpaired with complete data collected for sensory measures and brain MRI.

All participants provide written informed consent at each study visit, and the study protocol was approved by the Institutional Review Board of the National Institutes of Health Intramural Research Program^15^. All experiments were performed in accordance with U.S. Common Rule, 45 CFR 46.

### MRI-measured brain volumes

The primary study outcome is regional brain volumes measured by MRI. Brain MRI data were acquired using a 3T Philips Achieva scanner, with a T-1 volumetric scan magnetization prepared rapid acquisition with gradient echo scan (MPRAGE: repetition time = 6.8 ms, echo time = 3.2 ms, flip angle = 8°, image matrix = 256 × 256, 170 slices, pixel size = 1 × 1 mm, slice thickness = 1.2 mm; sagittal acquisition). Multi-atlas region Segmentation using Ensembles (MUSE) software was used to obtain anatomical labels and regional brain volumes^21,22^. There are 48 regional volumes of interest that broadly include total brain; gray and white matter; cerebellum; ventricular space; frontal, temporal, parietal, and occipital lobes; and specific regions within. Intracranial volume (ICV) was approximated using a template warping algorithm by estimating ICV residuals based on centering at age 70 years in the larger BLSA sample^23^.

### Sensory impairment

#### Vision

Vision was assessed in four ways: visual acuity, visual fields, contrast sensitivity, and stereo acuity. A participant was considered visually impaired if they had impairment in any of these features.

##### Visual acuity

Presenting visual acuity was based on better eye acuity from an ETDRS (Early Treatment of Diabetic Retinopathy Study) eye chart. Participants were given the ETDRS eye chart and asked to correctly read at least 3 letters out of 5 letters. Scores were calculated using the average of the logarithm 10 transfer of the minimum angle of resolution of the better eye. A score larger than 0.30 was defined as impaired visual acuity^24,25^.

##### Visual fields

Visual fields were measured with the area of vision that the participant can see using a single intensity visual field test. The test was given to each eye separately but combined to generate binocular fields using an algorithm. The visual fields were separated into the central field (56 points), the upper peripheral field (18 points), and the lower peripheral field (22 points). Scores were calculated by the total number of points missed (out of a total of 96 points) on the binocular visual field test, and visual field impairment was defined as a score greater than one standard deviation from the population mean^26^.

##### Contrast sensitivity

Contrast sensitivity was measured by the ability to discern between shades using a Pelli–Roboson chart positioned 1 m away, which is a 90 × 60 cm (36 × 24 inches) wall chart with 8 lines of letters with different contrasts. Each letter was 4.9 × 4.9 cm (2 × 2 inches) large, and there were 6 letters per line. The left 3 letters had more contrast than the right triplet. The contrast of the letters declined from the top to the bottom and from the left to the right of the chart. The top left had the highest contrast, 100%, and the bottom right had the lowest contrast, 0.6%. Scores were recorded as the logarithmic contrast sensitivity (1/contrast) of the line that at least 2 letters of the right triplet were correctly seen. Impairment was defined as the log of contrast sensitivity < 1.55, based on published population-based studies of older adults aged 60 years and older^24,25^.

##### Stereo acuity

Stereo acuity measures depth perception which was categorized using the Randot Stereo Vision Test^27,28^. The test booklet was presented upright in front of the participant who was asked to identify stereo images of decreasing depth differentials. The minimum depth differential in seconds of arcs was recorded (possible values: 800, 400, 200, 140, 100, 80, 60, 50, or 40), and if the value was less than or equal to 80, the participant was defined as impaired for stereo acuity^24,25^.

#### Vestibular function

Vestibular function was assessed by saccular function and semicircular canal function. A participant was considered impaired in vestibular function if they had impairment in any of these features.

##### Saccular function

Saccular function was measured using the cervical vestibular-evoked myogenic potential (cVEMP). During the test, participants sat on a chair and had electromyographic (EMG) electrodes placed on their sternocleidomastoid muscle and sternoclavicular junction bilaterally. The ground electrode was placed on the manubrium. Sound stimuli (500 Hz and 125 dB tone bursts) were delivered to evoke cervical myogenic potentials, which were recorded and normalized for background EMG. Saccular function impairment was defined if cVEMP was absent bilaterally^12^.

##### Semicircular canal function

Semicircular canal function was measured by vestibulo-ocular reflex (VOR) gain. VOR was determined using the Video Head Impulse Testing (VHIT), and VOR gain, the ratio of eye velocity to head velocity was determined using the EyeSeeCam system (Interacoustics, Eden Prarie, MN) by placing it at the same plane of the horizontal semicircular canals. During VHIT, the participant’s head was placed 30-degree down from the horizontal axis initially. Then, the participant was asked to fix their gaze on a 1.5-m away wall while the head was moved randomly 150–250 degrees per second in the horizontal plant toward the right or left. Each side was completed at least 10 times. The EyeSeeCam captured the eye and head velocity to calculate VOR gain. Semicircular canal function impairment was defined if VOR gain was smaller than 0.7^12^.

### Proprioception

Proprioception was defined by a threshold for perception of passive ankle movement. Participants’ bare feet were placed on two pedals at a neutral position. The right pedal has a motor that directs its movement, while the left pedal is moved by the participant. While blindfolded, the participant was asked to move their left foot to match the rotation angle of the right foot driven by the motor pedal^29^. There were four trials with a sequential pedal direction: plantarflexion, dorsiflexion, dorsiflexion, and plantarflexion. Proprioception impairment was defined as the average of best plantarflexion and best dorsiflexion was > 2.2 degrees, based on established thresholds for older adults^30^.

### Olfaction

Olfaction was measured with the number of correctly identified odors on the 16-item Sniffin’ Sticks Odor Identification test. Participants were randomly assigned to one of the two odor identification tests, A or B. Olfactory impairment was defined if the score was below the 10th percentile (8 for test A and 7 for test B) in the current sample^29,31^.

### Hearing

Hearing was measured with a speech frequency pure-tone average (PTA) at thresholds of 0.5, 1, 2, and 4 kHz. Participants were seated in a soundproof chamber under unaided conditions with an automated testing device (Virtual Equipment Co., Audiometer Model 320). The hearing score was both a categorical and continuous variable, with the former defined by the common-used WHO defined categories: normal (PTA < 25 dB), mild loss (25–40 dB), moderate loss (41–70 dB), and severe loss (> 70 dB). Participants were defined as hearing impaired if their PTA was $\ge$ 25 dBHL^32,33^.

### Covariates

Covariates in this study include age (years), sex, race, body mass index (BMI; kg/m^2^), usual gait speed (m/s), intracranial volume, and years of education. Age, sex, race, and years of educations collected via staff administered surveys. Usual gait speed (m/s) was measured during an 6 m overground walking test. BMI was calculated as weight (measured via digital scale) divided by height squared (measured via stadiometer). Intracranial volume (cm^3^) was also added as a covariate and estimated at age 70 years using linear mixed effects models. Each covariate has been separately associated with sensory impairments and brain volumes^15,23,34–37^.

### Statistical analysis

Independent *t*-tests or chi-square tests were performed to compare the demographic characteristics of the 208 study participants. Sensory impairments were analyzed dichotomously and included five types: vision, proprioceptive, vestibular, olfactory, and hearing impairment. Multisensory impairment (MSI) was defined when the individual had two or more co-occurring sensory impairments.

Each regional brain volume outcome was treated as a continuous variable using multivariable linear regression models. We analyzed cross-sectional associations of sensory impairments with brain volumes in four ways, differentiated based on how sensory impairments (SI) were operationalized. First, each sensory impairment (yes/no) was included as a predictor. Second, MSI was analyzed as a count (number of sensory impairments, ranging from 0 to 5). Third, MSI was analyzed as a categorical variable (MSI ≥ 2 sensory impairments vs. MSI < 2 sensory impairments) to capture co-occurring sensory impairments.

For each model comparing differences in brain volumes by categorical impairment variables, sensitivity analyses were conducted by redefining the reference of “no impairment” to those with no sensory impairment across any of the five sensory functions (n = 68). Though this study is exploratory and multiple comparisons were conducted, we added a correction for multiple comparison testing (n = 336 comparisons) using Benjamin–Hochberg false discovery rate (FDR)^38^.

All models were adjusted for age (years), sex, race, body mass index (BMI; kg/m^2^), years of education, usual gait speed (m/s), and intracranial volume. Two-tailed hypothesis testing with an alpha level = 0.05 was used to determine statistical significance. All statistical analysis was performed using Stata version 16.0 (Stata Corporation, College Station, TX; https://www.stata.com/stata16/).

## Results

Demographic characteristics of the final analytic sample (n = 208) by sensory impairment are summarized in Table 1. The overall mean age was 72.1 years with a standard deviation of 10.1 years (range 50–95 years) and 59% were women. Participants were well-educated (mean years of education 17.7 years, SD = 2.6 years) and functioning with a mean usual gait speed of 1.17 m/s (SD = 0.22 m/s).

**Table 1:** Characteristics of study population by composite sensory impairments (n = 208).

	Total	Vision impairment			Proprioception impairment				
	(n = 208)	Yes	No	p-value	Yes	No	p-value		
Participant, no. (%)	208	66 (31.7)	142 (68.3)		20 (9.6)	188 (90.4)			
Age (years), mean (sd)	72.1 (10.1)	76.1 (8.7)	70.2 (10.1)	< 0.001	74.3 (12.5)	71.9 (9.8)	0.312		
Women, no. (%)	122 (58.7)	38 (57.6)	84 (59.2)	0.830	10 (50.0)	112 (59.6)	0.408		
Body Mass Index (kg/m^2^), mean (sd)	27.3 (4.4)	26.3 (3.8)	27.8 (4.5)	0.028	27.4 (3.6)	27.3 (4.5)	0.935		
White, no. (%)	128 (61.5)	43 (65.2)	85 (59.9)	0.718	12 (60.0)	116 (61.7)	0.325		
Years of education, mean (sd)	17.7 (2.6)	18.2 (2.6)	17.5 (2.6)	0.106	17.8 (2.1)	17.7 (2.6)	0.914		
Usual pace gait speed (m/s), mean (sd)	1.17 (0.22)	1.11 (0.23)	1.20 (0.21)	0.005	1.09 (0.21)	1.18 (0.22)	0.069		
APOE e4 allele, no. (%)	50 (24.0)	13 (19.7)	37 (26.1)	0.318	3 (15.0)	47 (25.0)	0.320		

	Vestibular impairment			Olfaction impairment			Hearing impairment		
	Yes	No	p-value	Yes	No	p-value	Yes	No	p-value
Participant, no. (%)	38 (18.3)	170 (81.7)		28 (13.5)	180 (86.5)		93 (44.7)	115 (55.3)	
Age (years), mean (sd)	80.1 (7.3)	70.3 (9.7)	< 0.001	78.4 (6.9)	71.1 (10.1)	< 0.001	77.5 (8.3)	67.7 (9.1)	< 0.001
Women, no. (%)	20 (52.6)	102 (60.0)	0.404	13 (46.4)	109 (60.6)	0.158	45 (48.4)	77 (67.0)	0.007
Body Mass Index (kg/m^2^), mean (sd)	26.4 (4.4)	27.5 (4.4)	0.157	26.2 (4.0)	27.5 (4.4)	0.164	26.9 (4.1)	27.7 (4.6)	0.185
White, no. (%)	24 (63.2)	104 (61.2)	0.376	13 (46.4)	115 (63.9)	0.051	69 (74.2)	59 (51.3)	0.006
Years of education, mean (sd)	18.2 (2.4)	17.6 (2.7)	0.274	18.5 (2.0)	17.6 (2.7)	0.081	18.1 (2.7)	17.5 (2.5)	0.094
Usual pace gait speed (m/s), mean (sd)	1.08 (0.23)	1.20 (0.21)	0.003	1.18 (0.26)	1.17 (0.21)	0.838	1.14 (0.21)	1.20 (0.22)	0.054
APOE e4 allele, no. (%)	6 (15.8)	44 (25.9)	0.188	8 (28.6)	42 (23.3)	0.546	21 (22.6)	29 (25.2)	0.658

Independent *t*-test was performed for age, body mass index, years of education, and usual gait speed. Chi-square test was performed for sex, race, and APOE e4 allele.

Participants with vestibular impairment tended to be older on average than other groups (mean age 80.1 years, SD = 7.3 years), while participants with proprioceptive impairment tended to be younger than other groups on average (mean age 74.3 years, SD = 12.5 years). For all sensory impairments except proprioception, the mean age was higher for those who were impaired than the unimpaired. Those with hearing impairments tended to be men (p = 0.006) and White (p = 0.007). Participants with impaired vision and vestibular function had significantly slower usual gait speed than participants without these impairments.

The distribution of sensory impairments by number and type are shown in Table 2. Among 208 participants, 138 participants (66.5%) had no or only one sensory impairment. The most prevalent sensory impairment was hearing (44.7%) whereas 9.6% had proprioceptive impairment. Forty-four participants (21.1%) had two sensory impairments with vision and hearing the most common pair (n = 20, 9.6%), followed by vestibular and hearing (n = 8, 3.8%). For multiple co-occurring impairments, nineteen participants (9.1%) had three, five participants (2.4%) had four and two participants had five.

**Table 2:** Distribution of multisensory impairments (n = 208).

	Numbers of participants
Numbers of sensory impairment	
0	68 (33.0%)
1	70 (33.5%)
2	44 (21.1%)
3	19 (9.1%)
4	5 (2.4%)
5	2 (1.0%)
Combinations of two impairments	
VS + H	20 (9.6%)
VES + H	8 (3.8%)
SML + H	4 (1.9%)
VS + SML	3 (1.4%)
VS + VES	3 (1.4%)
VS + PROP	2 (1.0%)
VES + SML	2 (1.0%)
PROP + H	2 (1.0%)
VES + PROP	0 (0.0%)
PROP + SML	0 (0.0%)
Combinations of three impairments	
VS + SML + H	6 (2.9%)
VS + VES + H	5 (2.4%)
VS + PROP + H	2 (1.0%)
VES + SML + H	2 (1.0%)
VS + VES + PROP	1 (0.5%)
VS + VES + SML	1 (0.5%)
VES + PROP + H	1 (0.5%)
PROP + SML + H	1 (0.5%)
VES + PROP + SML	0 (0.0%)
VS + PROP + SML	0 (0.0%)
Combinations of four impairments	
VS + VES + SML + H	3 (1.4%)
VS + VES + PROP + H	2 (1.0%)
VS + VES + PROP + SML	0 (0.0%)
VS + PROP + SML + H	0 (0.0%)
VES + PROP + SML + H	0 (0.0%)

*VS* visually impaired, *H* hearing impaired, *VES* vestibular impaired, *SML* olfactory impaired, *PROP* proprioceptive impaired.

### Single sensory impairment and brain volumes

Participants with vision impairment had lower frontal gray matter volume ($\beta$ = − 3.00 cm^3^, SE = 1.45, p = 0.041) and inferior temporal gyrus volume ($\beta$ = − 0.89 cm^3^, SE = 0.24, p < 0.001) compared to participants without vision impairment in fully adjusted models, the latter association remained statistically significant after the FDR analysis (Table 3). Participants with proprioceptive impairment had higher ventricular space ($\beta$ = 10.10 cm^3^, SE = 3.84, p = 0.009) and frontal lobe white matter ($\beta$ = 5.10 cm^3^, SE = 2.53, p = 0.046), and caudate volumes ($\beta$ = 0.38 cm^3^, SE = 0.16, p = 0.019), and lower entorhinal cortex volume ($\beta$ = − 0.26 cm^3^, SE = 0.10, p = 0.008) than participants without proprioceptive impairment. Participants with vestibular impairment had lower superior frontal gyrus volume ($\beta$ = − 0.94 cm^3^, SE = 0.38, p = 0.015), lower orbitofrontal gyrus volume ($\beta$ = − 0.91 cm^3^, SE = 0.43, p = 0.033), lower superior parietal lobe volume ($\beta$ = − 1.00 cm^3^, SE = 0.29, p = 0.001), lower superior occipital gyrus volume ($\beta$ = − 0.35 cm^3^, SE = 0.17, p = 0.047), and lower inferior occipital gyrus volume ($\beta$ = − 0.57 cm^3^, SE = 0.23, p = 0.016) than those without vestibular impairment. The lower superior parietal lobe volume association remained statistically significant after the FDR analysis. Participants with olfactory impairment had lower orbitofrontal gyrus volume ($\beta$ = − 0.94 cm^3^, SE = 0.50, p = 0.047) and lower posterior cingulate gyrus volume ($\beta$ = − 0.34 cm^3^, = 0.16, p = 0.035).

**Table 3:** Differences in mean regional brain volumes (cm^3^) for each separate sensory impairment^.

	Vision impairment	Proprioceptive impairment	Vestibular impairment	Olfactory impairment	Hearing impairment
	Beta coefficient (SE)				
Cerebellum	1.867 (1.593)	− 4.054 (2.290)	− 0.475 (1.960)	1.579 (2.273)	− 0.162 (1.677)
Total brain	0.940 (6.412)	16.157 (9.613)	− 10.873 (7.914)	6.196 (8.725)	2.909 (6.763)
Ventricular space	0.473 (2.584)	10.095 (3.836)**	− 1.685 (3.203)	3.900 (3.510)	0.634 (2.726)
Gray matter	− 4.846 (3.846)	− 1.238 (5.829)	− 6.684 (4.765)	0.235 (5.260)	0.628 (4.074)
White matter	5.145 (3.676)	7.734 (5.549)	− 2.478 (4.577)	1.873 (5.030)	1.500 (3.896)
Frontal lobe	− 0.604 (2.544)	7.129 (3.808)	− 2.322 (3.151)	− 2.155 (3.463)	1.809 (2.681)
Temporal lobe	− 0.932 (1.378)	− 0.377 (2.083)	− 1.798 (1.706)	0.872 (1.879)	1.173 (1.454)
Parietal lobe	− 0.033 (1.463)	0.720 (2.208)	− 2.657 (1.805)	0.910 (1.992)	− 0.107 (1.544)
Occipital lobe	− 0.136 (1.168)	1.977 (1.758)	− 1.520 (1.445)	0.691 (1.591)	− 1.024 (1.230)
Frontal gray matter	− 2.997 (1.454)*	2.030 (2.213)	− 2.194 (1.815)	− 1.839 (1.997)	0.723 (1.549)
Temporal gray matter	− 1.710 (0.902)	− 1.390 (1.370)	− 0.886 (1.126)	− 0.177 (1.239)	1.042 (0.957)
Parietal gray matter	− 1.018 (0.881)	− 0.333 (1.334)	− 2.057 (1.087)	0.361 (1.204)	− 0.400 (0.932)
Occipital gray matter	− 0.571 (0.801)	0.574 (1.210)	− 0.946 (0.992)	0.250 (1.093)	− 0.657 (0.845)
Frontal white matter	2.393 (1.687)	5.098 (2.534)*	− 0.128 (2.103)	− 0.316 (2.310)	1.086 (1.787)
Temporal white matter	0.778 (0.877)	1.014 (1.325)	− 0.912 (1.088)	1.049 (1.195)	0.131 (0.927)
Parietal white matter	0.985 (0.874)	1.054 (1.322)	− 0.600 (1.087)	0.549 (1.194)	0.294 (0.925)
Occipital white matter	0.435 (0.535)	1.403 (0.803)	− 0.574 (0.663)	0.441 (0.729)	− 0.366 (0.565)
Superior frontal gyrus	− 0.578 (0.309)	0.381 (0.470)	− 0.938 (0.381)**	− 0.629 (0.422)	0.408 (0.327)
Middle frontal gyrus	− 0.397 (0.393)	0.454 (0.594)	0.044 (0.488)	0.152 (0.536)	0.350 (0.415)
Inferior frontal gyrus	− 0.199 (0.218)	0.307 (0.329)	− 0.091 (0.271)	0.070 (0.298)	0.018 (0.231)
Medial frontal cortex	− 0.017 (0.069)	0.023 (0.105)	− 0.113 (0.086)	− 0.003 (0.095)	− 0.010 (0.073)
Orbitofrontal gyrus	− 0.618 (0.345)	0.338 (0.524)	− 0.914 (0.426)*	− 0.936 (0.469)*	0.139 (0.367)
Precentral gyrus	− 0.298 (0.258)	0.187 (0.391)	0.338 (0.320)	− 0.228 (0.353)	0.021 (0.273)
Postcentral gyrus	− 0.030 (0.266)	− 0.355 (0.401)	− 0.412 (0.329)	0.127 (0.363)	0.069 (0.281)
Superior parietal lobe	− 0.239 (0.241)	− 0.046 (0.365)	− 0.999 (0.291)**^#^	− 0.205 (0.329)	0.160 (0.255)
Supramarginal gyrus	− 0.169 (0.224)	0.280 (0.337)	− 0.394 (0.276)	0.111 (0.305)	0.038 (0.236)
Angular gyrus	− 0.255 (0.276)	− 0.526 (0.416)	0.316 (0.342)	0.196 (0.376)	− 0.399 (0.290)
Precuneus	− 0.346 (0.320)	0.233 (0.485)	− 0.529 (0.397)	0.202 (0.438)	− 0.300 (0.338)
Superior temporal gyrus	0.125 (0.194)	0.159 (0.293)	− 0.233 (0.240)	− 0.190 (0.264)	0.289 (0.204)
Middle temporal gyrus	− 0.328 (0.340)	− 0.923 (0.511)	− 0.242 (0.423)	− 0.122 (0.465)	− 0.250 (0.359)
Inferior temporal gyrus	− 0.889 (0.240)***^#^	− 0.199 (0.374)	− 0.040 (0.308)	− 0.109 (0.338)	0.410 (0.260)
Hippocampus	− 0.086 (0.092)	0.059 (0.139)	− 0.118 (0.114)	-0.046 (0.125)	− 0.162 (0.096)
Parahippocampus	− 0.069 (0.091)	− 0.060 (0.137)	− 0.132 (0.112)	0.010 (0.124)	− 0.044 (0.096)
Entorhinal cortex	− 0.089 (0.066)	− 0.264 (0.098)**	− 0.104 (0.082)	− 0.115 (0.090)	− 0.034 (0.070)
Amygdala	− 0.018 (0.030)	− 0.034 (0.045)	− 0.020 (0.037)	− 0.014 (0.041)	− 0.050 (0.031)
Fusiform gyrus	− 0.151 (0.218)	− 0.177 (0.329)	0.029 (0.271)	0.145 (0.297)	0.294 (0.229)
Superior occipital gyrus	0.089 (0.142)	0.030 (0.214)	− 0.348 (0.174)*	0.051 (0.193)	0.141 (0.149)
Middle occipital gyrus	− 0.306 (0.179)	0.156 (0.272)	0.052 (0.224)	− 0.083 (0.246)	− 0.156 (0.190)
Inferior occipital gyrus	− 0.108 (0.192)	− 0.116 (0.289)	− 0.567 (0.234)*	− 0.014 (0.261)	− 0.234 (0.202)
Occipital pole	− 0.108 (0.142)	0.282 (0.214)	0.190 (0.176)	− 0.071 (0.194)	0.002 (0.150)
Cuneus	0.146 (0.178)	0.018 (0.269)	0.147 (0.221)	0.120 (0.243)	-0.058 (0.188)
Anterior cingulate gyrus	− 0.064 (0.160)	0.470 (0.240)	0.024 (0.199)	− 0.176 (0.218)	0.222 (0.168)
Posterior cingulate gyrus	− 0.143 (0.117)	0.150 (0.177)	0.024 (0.146)	− 0.336 (0.158)*	-0.089 (0.124)
Middle cingulate gyrus	− 0.090 (0.137)	0.205 (0.206)	− 0.093 (0.169)	0.055 (0.186)	0.163 (0.144)
Caudate	0.170 (0.107)	0.378 (0.160)*	0.063 (0.133)	0.254 (0.145)	0.084 (0.113)
Globus pallidus	0.024 (0.040)	0.0130 (0.060)	− 0.022 (0.050)	− 0.020 (0.055)	0.065 (0.042)
Putamen	0.119 (0.128)	0.189 (0.193)	0.159 (0.159)	0.267 (0.174)	0.085 (0.135)
Thalamus	− 0.108 (0.144)	0.321 (0.217)	0.091 (0.179)	0.186 (0.196)	− 0.026 (0.152)

All are multivariable linear regression models adjusted for age, sex, race, BMI (body mass index), icv70 (intracranial volume at age 70 years old), and years of education.

^The reference group for each column is no impairment in that specific sensory function.

*p < 0.05; **p < 0.01; ***p < 0.001.

^#^The association remained statistically significant after the FDR analysis.

The associations between vision impairment and inferior temporal gyrus ($\beta$ = − 1.33 cm^3^, SE = 0.41, p = 0.002) and proprioception impairment and entorhinal cortex ($\beta$ = − 0.40 cm^3^, SE = 0.18, p = 0.035) were robust to sensitivity analyses comparing each sensory impairment with participants who had no sensory impairments (n = 68). The associations between proprioception impairment with ventricular space (p > 0.05) and olfactory impairment with posterior cingulate gyrus (p > 0.05) were not robust to sensitivity analyses. However, the other associations had similar magnitude and directionality as reported above but did not reach statistical significance (p > 0.05).

### Count of sensory impairments and brain volumes

Each additional sensory impairment was associated with lower mean volume of the orbitofrontal gyrus ($\beta$ = − 0.35 cm^3^, SE = 0.17, p = 0.04) and in the entorhinal cortex ($\beta$ = − 0.09 cm^3^, SE = 0.03, p = 0.006) in fully adjusted models (Table 4, first column). Only the association with entorhinal cortex remained statistically significant after FDR correction. There was also a higher mean volume in the caudate ($\beta$ = 0.14 cm^3^, SE = 0.05, p = 0.006) and in the putamen ($\beta$ = 0.13 cm^3^, SE = 0.06, p = 0.043) of the basal ganglia though none of the results remained statistically significant after FDR correction.

**Table 4:** Associations between MSI (as a count and in categories) and mean regional brain volumes (cm^3^) (n = 208).

	Continuous SI ranging from 0 to 5 impairments	Categorical MSI (MSI $\ge$ 2 vs. MSI < 2)
		Beta coefficient (SE)
Cerebellum	0.055 (0.762)	− 1.382 (1.762)
Total brain	1.679 (3.158)	− 5.286 (7.140)
Ventricular space	1.574 (1.269)	1.747 (2.879)
Gray matter	− 2.197 (1.897)	− 8.332 (4.265)
White matter	2.255 (1.813)	1.605 (4.117)
Frontal lobe	0.359 (1.254)	− 1.429 (2.835)
Temporal lobe	− 0.180 (0.680)	− 1.053 (1.537)
Parietal lobe	− 0.255 (0.721)	− 2.722 (1.620)
Occipital lobe	− 0.195 (0.576)	− 0.216 (1.302)
Frontal gray matter	− 0.941 (0.721)	− 3.197 (1.622)*
Temporal gray matter	− 0.499 (0.447)	− 1.334 (1.010)
Parietal gray matter	− 0.648 (0.433)	− 2.510 (0.970)*
Occipital gray matter	− 0.338 (0.395)	− 0.579 (0.893)
Frontal white matter	1.300 (0.831)	1.768 (1.886)
Temporal white matter	0.319 (0.433)	0.282 (0.980)
Parietal white matter	0.393 (0.431)	− 0.211 (0.978)
Occipital white matter	0.142 (0.264)	0.363 (0.597)
Superior frontal gyrus	− 0.241 (0.153)	− 1.014 (0.340)**
Middle frontal gyrus	0.055 (0.194)	− 0.15 (0.439)
Inferior frontal gyrus	− 0.017 (0.108)	− 0.118 (0.243)
Medial frontal cortex	− 0.022 (0.034)	0.015 (0.077)
Orbitofrontal gyrus	− 0.351 (0.170)*	− 0.914 (0.382)**
Precentral gyrus	− 0.024 (0.128)	− 0.129 (0.289)
Postcentral gyrus	− 0.078 (0.131)	− 0.356 (0.296)
Superior parietal lobe	− 0.213 (0.118)	− 0.679 (0.265)**
Supramarginal gyrus	− 0.051 (0.110)	− 0.456 (0.248)
Angular gyrus	− 0.130 (0.136)	− 0.302 (0.307)
Precuneus	− 0.182 (0.158)	− 0.740 (0.354)*
Superior temporal gyrus	0.049 (0.096)	− 0.0001 (0.216)
Middle temporal gyrus	− 0.287 (0.167)	− 0.548 (0.378)
Inferior temporal gyrus	− 0.168 (0.122)	− 0.367 (0.276)
Hippocampus	− 0.075 (0.045)	− 0.114 (0.102)
Parahippocampus	− 0.052 (0.045)	− 0.119 (0.101)
Entorhinal cortex	− 0.089 (0.032)**^#^	− 0.081 (0.074)
Amygdala	− 0.024 (0.015)	− 0.043 (0.033)
Fusiform gyrus	0.032 (0.108)	− 0.175 (0.243)
Superior occipital gyrus	0.007 (0.070)	− 0.038 (0.158)
Middle occipital gyrus	− 0.094 (0.089)	− 0.183 (0.201)
Inferior occipital gyrus	− 0.181 (0.094)	− 0.211 (0.213)
Occipital pole	0.025 (0.070)	− 0.065 (0.159)
Cuneus	0.064 (0.088)	0.197 (0.198)
Anterior cingulate gyrus	0.064 (0.079)	0.106 (0.179)
Posterior cingulate gyrus	− 0.078 (0.058)	− 0.173 (0.131)
Middle cingulate gyrus	0.028 (0.067)	0.006 (0.152)
Caudate	0.143 (0.052)**	0.213 (0.119)
Globus pallidus	0.015 (0.020)	0.017 (0.045)
Putamen	0.128 (0.063)*	0.173 (0.143)
Thalamus	0.041 (0.071)	0.045 (0.161)

*SI* sensory impairment, *MSI* multisensory impairment.

All are multivariable linear regression models adjusted for age, sex, race, BMI (body mass index), icv70 (intracranial volume at age 70 years old), and years of education.

*p < 0.05; **p < 0.01; ***p < 0.001.

^#^The association remained statistically significant after the FDR analysis.

### MSI and brain volumes

In fully adjusted models, participants with two or more co-occurring sensory impairments (n = 70, 34%) had lower mean volume in the frontal gray matter ($\beta$ = − 3.20 cm^3^, SE = 1.62, p = 0.05), parietal lobe gray matter ($\beta$ = − 2.51 cm^3^, SE = 0.97, p = 0.01), superior frontal gyrus ($\beta$ = − 1.01 cm^3^, SE = 0.34, p = 0.003), orbitofrontal gyrus ($\beta$ = − 0.91 cm^3^, SE = 0.38, p = 0.018), superior parietal lobe ($\beta$ = − 0.68 cm^3^, SE = 0.27, p = 0.011), and precuneus ($\beta$ = − 0.74 cm^3^, SE = 0.35, p = 0.038) compared to those with less than two sensory impairments (Table 4, second column). None of the results remained statistically significant after FDR correction.

These results were not robust when changing the reference to participants with no sensory impairments. However, the sensitivity analyses showed that those with two or more sensory impairments had lower amygdala volume ($\beta$ = − 0.04 cm^3^, SE = 0.02, p = 0.04) compared to those with no sensory impairments.

### Sensory impairment patterns with brain volumes

For participants with two co-occurring sensory impairments (MSI = 2), there were 8 observed combinations; for participants with three sensory impairments (MSI = 3), there were 8 observed combinations; for participants with four types of sensory impairments (MSI = 4), there were 2 observed combinations (Table 2).

## Discussion

This study characterized cross-sectional associations between co-occurring sensory impairments and brain volumes in cognitively unimpaired adults aged 50 years and older. Combinations of MSI that include vision, proprioceptive, and/or vestibular impairments were associated with lower brain volumes in the frontal gray matter, superior frontal gyrus, orbitofrontal gyrus, superior parietal lobe, and entorhinal cortex regions. Further, there is a possibly that MSI is uniquely associated with the lower amygdala volume. Oppositely, MSI was associated with higher volumes in the basal ganglia region, suggesting a possible compensatory mechanism within this region as the brain attempts to adapt to brain atrophy in other regions. Collectively, these results highlight potential associations between MSI and brain structure so that future longitudinal research focusing on these regions may be performed to understand the temporality of these associations.

It is unclear the specific sensory impairments that drive the detected association between MSI and lower volume in the orbitofrontal gyrus. However, in the single sensory impairment analyses, vestibular and olfactory impairment separately were significantly associated with lower orbitofrontal gyrus volume. This suggests that a combination of vestibular and/or olfactory impairments may reflect or result from lower orbitofrontal gyrus volume, but the sample with both vestibular and olfactory impairment is too small to produce stable estimates. This is supported by animal studies showing that neural pathways between sensory cortices and the orbitofrontal gyrus exhibit diminished numbers of neural connections in animals with sensory impairments^39,40^. It is important to acknowledge that this finding was not robust when accounting for multiple testing.

The observed association between MSI and lower volume in the entorhinal cortex is consistent with known inputs from the vestibular system into the entorhinal cortex, which contains a neuronal population called grid cells which are involved in spatial navigation^41^. Indeed, vestibular impairment has been associated with entorhinal cortex atrophy in prior work^42^. These findings are also consistent with a previous study in rats, which performed deafferentation injury surgeries on the entorhinal cortex of rats’ brains and assessed the rats’ performance using sensory integration testing. By analyzing histological brain slides and performance scores, researchers concluded that rats with lesions in the entorhinal cortex showed sensory integration deficits and behavioral change^43^. This suggests that the entorhinal cortex plays a role in multisensory function. Future research into the biological mechanisms behind the link between MSI and both the orbitofrontal gyrus and entorhinal cortex in humans is warranted.

In sensitivity analyses comparing MSI versus no sensory impairment, a possible unique association was revealed between two or more sensory impairments and lower amygdala volumes. If this association is true, this is consistent with the amygdala’s role in the processing of sensory information^44^. However, this finding did not remain statistically significant when accounting for multiple testing. Without replication of this finding in larger studies, this relationship is likely spurious since it was not detected in the main analysis.

MSI and proprioceptive impairment alone were associated with a higher mean volume in the basal ganglia region in the main analysis but did not remain statistically significant after multiple testing correction. Still, a possible explanation for this potential finding is that the basal ganglia region may play a compensatory role in how the brain adapts to MSI. The basal ganglia are involved in both motor control and cognitive function^45^ and are thought to work with cortical regions in executing stereotyped motor and cognitive actions under cortical volitional control. Conceivably, with the loss of sensory-driven cortical control, “implicit” behaviors and actions stored in the basal ganglia are increasingly activated or are lost more slowly than other brain regions. A previous study using a smaller sample of BLSA participants also demonstrated that worse vestibular function was related to higher volumes of the basal ganglia region, the caudate and putamen, specifically^42^. Also, this finding might coincide with the basal ganglia tending to be last to atrophy and lose function according to the “first in, last out” principle^46^. Collectively, these findings suggest the possibility that proprioceptive and/or vestibular dysfunction may trigger a higher utilization of the basal ganglia, which indirectly mitigates this region’s atrophy with age.

Two or more co-occurring sensory impairments were associated with lower volumes in the gray matter of the frontal and parietal lobes, in the superior frontal gyrus and superior parietal lobe, and the precuneus. Our findings suggest that these gray matter associations may be driven by vision impairment and the frontal and parietal lobe associations driven by vestibular impairment. However, caution in interpreting these exploratory findings should be noted since the prevalence of various patterns was low and subject to type 1 error due to multiple testing and did not remain statistically significant after accounting for multiple testing.

There are limitations to acknowledge. The first limitation is the small sample size, that was defined with participants who had all five sensory measures plus complete brain MRI scans. Second, the generalizability of the findings is narrow because BLSA participants tend to have higher education and better health than the general older adult population. Third, the prevalence of MSI with three or more sensory impairments observed was low, yielding low statistical power. Fourth, the cross-sectional design does not account for temporality and thus the direction of association is unclear. Fifth, multiple statistical tests were performed, increasing the likelihood of type 1 error. Though this was an exploratory study, we performed FDR analyses in the sensitivity analyses to address issues with multiple comparison testing. Yet, when doing so might increase type II error that reduces the detection of true associations.

This study has multiple strengths. First, sensory impairments were comprehensively measured across a large sample of older adults. Second, brain volumes were quantified via brain MRI scans. Third, this study was able to describe specific patterns of MSI and explore their relationships to brain volumetric measures.

In conclusion, this study found that higher numbers of sensory impairments were linked to higher ventricular volumes and lower brain volumes, primarily in the superior frontal gyrus, orbitofrontal gyrus, and precuneus. In contrast, those living with MSI had higher volumes in the basal ganglia regions. Future research is needed to explore longitudinal associations to evaluate whether multiple sensory impairments lead to accelerated brain atrophy and whether there are some brain regions may be preserved or activated (i.e., the basal ganglia) in response, and assess whether these relationships are linked to cognitive outcomes. Also, leveraging machine learning approaches (e.g., random forest) using MSI information to detect or predict changes in brain volumes are warranted. Such work may help provide mechanistic insights linking sensory impairments with aging brain and detection of cognitive decline. Replication is needed given the exploratory nature of the findings and the possibility of false discovery.

## Data Availability

Because of the sensitive nature of the data collected for this study, requests to access the data set from qualified researchers trained in human subject confidentiality protocols may be sent to the Intramural Research Program of the National Institute on Aging at https://blsa.nih.gov.
